# Leçons faisant Partie du Cours de Médecine Legale

**Published:** 1821-09

**Authors:** 


### CRITICAL ANALYSES

OF

### RECENT PUBLICATIONS, IN THE DIFFERENT BRANCHES OF MEDICINE AND SURGERY,

### In the Literature of Foreign Nations.

Πατρίδες ἄρα
Ανδράσιν, ὦ πατραῖς ἄνδρες, ἀγαλλόμεθα.

*Leçons faisant Partie du Cours de Médecine Legale de* M. Orfila, Professeur de Médecine légale à la Faculté de Médecine de Paris, &c. &c. 8vo. pp. 487.

[In continuation from page 255.]

THE poisonous champignons and mushrooms appertain to the genera *amanita* and *agaricus*.

The characters of the genus amanita are: a champignon springing from a purse or *volva;* the cap furnished, at its inferior surface, with radiated lamellæ, and supported by a stalk having a more or less large tuberosity at its base.

The species of this genus are—

1. The *agaricus muscarius* of Linnæus; the particular characters of which are as follows: The cap is from five to seven inches in diameter; it is at first convex, and afterwards becomes nearly horizontal; the scarlet hue of its upper surface is deepest in the centre; it is a little radiated at its margin, and almost always spotted with white tubercles or warts, which are the remnants of the *volva;* the stalk, about two inches and a half or three inches in length, is white, firm, cylindrical, except at its base, where it is tumid; the gills are white, unequal, and covered, when but young, with a membrane which afterwards retracts around the stalk and forms its collar. The volva is incomplete,—that is to say, it does not completely cover the cap on its first springing from the ground, and it forms several scales along the stalk. This champignon is very common in the northern part of Europe.

There is a variety of the *amanita* (the *aurantica*), which is sometimes eaten, and is distinguishable from the foregoing,—1, because, when young, it is enveloped in the volva, which gives it some resemblance to an egg; 2, by the orange colour of the cap, which, besides, is not spotted with white tubercles; 3, by the gills, which are of a yellowish colour.

Another species of this genus is the *amanita venenosa* of Persoon, (which comprises the varieties *agaricus bulbosus* and *agaricus bulbosus vernus* of Bulliard.) Its characters are a whitish, sulfurine, or greenish, colour; a bulbous stalk, surrounded at its base by a volva, which covers the cap before its development, and on which there remains some fragments which are large and irregular in form towards its margin, but smaller and polyhedral at the middle; there is on the stalk a somewhat large, thick, and often rugged, ring or collar. The gills are white, and always preserve this colour. The cap is convex, fleshy, three or four fingers' breadth in diameter, and but rarely devoid of tubercles: its odour is virous, and very strong; its taste is acrid and styptic, especially when it has been chewed for a few instants.

The characters of the first variety (the *amanita bulbosa alba* of Persoon,) are as follows: it is entirely white, though sometimes a little yellowish at its summit; the cap, which was at first convex, becomes at length concave, because its margin turns upwards as it becomes old: its gills are numerous, and divided into lamellæ continuous throughout its semi-diameter, and such as are subdivided into others of more partial extent. It may be distinguished from the eatable champignon, because the latter has neither volva nor tuberosity at the base of its stalk; and because this may be easily peeled, has a very irregular collar, which looks as if it had been gnawed at its margin; the superior surface of the cap is, besides, dry, and its gills are always of a roseate colour, at first but pale, afterwards deeper, and at length of a dark brown; whilst the gills of the poisonous species are always white. The poisonous species is very common in woods, and has often been productive of serious accidents, from its having been confounded with that which is eatable.

The second variety (the *amanita citrina* of Persoon, and *agaricus bulbosus* of Bulliard,) may be recognized by the following characters. The cap, and the ring or collar, are of a pale citron colour; the stalk, three or four inches in length, is bulbous, and slightly striated at its summit. It is found in abundance in autumn, amidst the dried leaves in the shaded parts of woods or coppices.

The third variety is the *amanita viridis*, (or *agaricus bulbosus* of Bulliard:) its characters are, a cap, almost always rough, without any fragments of a volva; the tuberosity at the base of the stalk is rounder than in the two foregoing varieties. It is of a pale sap-green colour, though sometimes it is of an olive or greyish hue: it is larger than either of the foregoing varieties. It is found in autumn, in the thicker parts of woods.

The *hypophillum maculatum* is a champignon of a white or greyish hue, the size of which is various, but which is ordinarily three or four inches in height. Its stalk has a collar of greyish pellicles; its gills and stalk, with the bulb at its foot, are perfectly white. Its surface is viscous. The cap is soft, three or four inches in diameter, hardly of a fleshy consistence, slightly radiated, is easily peeled, and apt to split. Its gills are interspersed with only portions of radiated laminæ about the margin, and their sharp edge is a little serrated: they are inserted, in a circular manner, in an axis which does not touch the stalk, and are covered, when young, with a film which afterwards separates from them and falls round the stalk, forming the collar already noticed. The stalk, at first full, becomes at length, as well as the bulb, in a great degree hollow.

The *hypophillum albo-citrinum*, is a champignon of the middling size, of a very regular form, sometimes of a white hue tinged with yellow, with spots here and there of a deeper yellow or earthy colour; in others, the cap is smooth, and of a clear white or pale yellow colour. Its bulb at the foot of the stalk is large, prominent, and round. The stalk is straight and cylindrical; white, or variously coloured, as is the case with champignons generally: it is at first full; it then becomes partly hollow, and expands at its insertion into the cap, with which it seems to be confounded. The cap is circular, and more or less moist. The gills are white; their sharp edges are smooth and uniform; almost all of them are of equal length, with the exception of some small laminous portions which are situate near the margin, and which seemed to be attached, at their bases, to the complete laminæ, as it were by little bridles. The gills are inserted into an axis which does not touch the stalk. It has constantly a collar, which was originally a film covering the gills.

The *hypophillum tricuspidatum* is five or six inches in height, white, with gills of a colour verging to green. The cap is regularly circular, thickly spotted with equal triangular points, of a pyramidal figure, of a dirty-white hue, and strongly adherent by their basis to the film which envelops the cap. The gills are ordinarily covered by a sort of dust, and with a film which is inserted on the stalk and serves it for a collar. The stalk is white, cylindrical, full, with a bulb at its foot, which becomes hollow as well as the stalk itself.

The *hypophillum rapula* is a small champignon, the cap of which is of a nut colour on its upper surface, and is studded with a multitude of unequal points, similar to those of an ordinary rasp, and of a deeper colour than the cap itself. The laminæ of the gills are very thin, close-set, white, covered at first by a tender film, which is soon torn into several pieces, and which is at length entirely effaced. The stalk is white, and filled with a pithy substance.

The *hypophillum anguineum* is a tall champignon; its cap is of a conical form, of a mouse-grey colour, and of a satinous appearance on its surface; the gills are nearly white. The base of the cap is about an inch and a half in diameter, and its interior structure seems to be constituted of little grey grains, which make it appear of an ash colour, at a distance, when the cap has been cut open. The stalk is four or five inches in length, of a dirty-white colour; it is filled with very white pulp, and bears at its foot the fragments of a fine envelopment which had covered the whole of the champignon.

The *Maltese cross (hypophillum crux melitensis* of Paulet,) is a champignon whose cap is slit into five or six equal parts, so that it has the figure of the cross from which it derives its vulgar appellation. There is in the centre of the cross a button, a little raised from the rest and regularly circumscribed. The lobes are about two lines in thickness. The laminæ of the gills are almost equal in size, and, as well as the surface of the cap, of a pale flesh-colour. They are inserted into a sort of axis, without touching the stalk. The stalk is straight, provided with a collar, three or four inches in length, at first full, but becoming at length hollow. The collar and volva are of a clear white colour.

The *hypophillum pudibundum*, has a cap with the central part elevated into a point, which at length is effaced to give place to a cavity. It is white, but its substance, as well as the juice it contains, assumes a carmine colour when it has been for some time exposed to the air. Its gills are white, and of equal dimensions. Its stalk, which is a continuation of the substance of the cap, is cylindrical, and full of a pulpy matter.

The last we have to notice of this genus is the *hypophillum pellitum*, which, as figured by Mr. Paulet, has a large cylindrical stalk, a little tumefied at its lower part; it is about six inches in height; it has a circular collar about its upper part, which is membranaceous and unequally fringed at its floating margin. This stalk is of a dirty-white colour. The cap is unequally convex, about six inches in diameter; its margin is furrowed; it is of a yellowish-grey colour on its upper surface, and is covered with little irregular flakes, of a deeper colour, which seem to be the remains of the volva in which the whole of the champignon had been enveloped at its first appearance.

We have given Prof. Orfila's particular descriptions of the poisonous mushrooms, because they comprise information that may be of considerable utility to medical practitioners, and which cannot be elsewhere obtained, with so much accuracy. Of the species of the genus *agaricus* we need say but little, as there are but very few of them which are likely to be mistaken for eatable mushrooms. It is possible, however, that the *agaricus necator*, *agaricus piperatus*, and the *agaricus urens*, when young, may be gathered for the latter by some of the ignorant persons who so commonly employ themselves in collecting them. The agaricus urens has, when young, a convex cap, which afterwards becomes flat, and then concave in the centre. The margin of the cap is curled inwards, is fringed, and has, as well as the general surface, a velvet-like appearance. Its upper surface is sometimes marked with concentric zones, the diameter of which does not in general exceed three inches, of a pale, fleshy, or tan colour, becoming paler towards the margin of the cap. The stalk is cylindrical, full, bare, thick, and at most three or four inches in length. The gills are of a pale-yellow colour, and the small number of laminæ which constitute them form a sort of axle at their insertion into the stalk. It is very common in woods, amongst graminous vegetables, in summer and autumn.

The agaricus piperatus has a very white cap, which is very round when the fungus is young; it afterwards takes the form of a tunnel, and its margins, which are a little cottony, become unequally defined. The stalk is full, short, and thick. The laminæ forming its gills, sometimes entirely, at others but partially, run throughout its whole semi-diameter; and they may be either but few or very numerous. They are of a white colour when the champignon is young, but assume a straw colour as it becomes old. It is common in woods, in summer and autumn.

The agaricus urens has a cap which is at first convex, afterwards flat, of a regular form, from an inch and a half to two inches in diameter, a pale and dirty yellow colour. The laminæ of the gills are red, unequal, and those which are entire do not quite reach the stalk. The stalk is cylindrical, four or five inches in length, a little tuberous and villous at its base, bare, full, continuous with the substance of the cap, of a pale-yellow or earthy colour, a little striated with red. This champignon springs from dead leaves.

In addition to the observations of Prof. Orfila, we may remark, in a general allusion, that all mushrooms of a deep or brown colour should be avoided, as meriting suspicion of their wholesomeness: the species, too, which have a collar or volva, and those of which the juice becomes blue or green when exposed to the air, are highly poisonous; as are those also which have a peppery savour. Old mushrooms or champignons are more dangerous than young ones. Those which have a milky juice are generally poisonous.

The symptoms of poisoning by those funguses are, in general, as follows. The patients suffer, in the first place, gripings, nausea, and vomiting and purging; a sense of intense heat in the bowels then comes on, with great langour, and constant and very severe pain. Then ensue cramps; convulsions, general or partial; and inextinguishable thirst; the pulse becomes small, hard, contracted, and very frequent. If the malady continues to make further progress, sometimes vertigo, at others a dull delirium, or stupor, takes place. These symptoms are interrupted only by pains and convulsions. In certain cases there is no stupor, and the pains are extremely intense and the convulsions most violent. Fainting and cold sweats precede the occurrence of death, whilst the patient preserves the use of his senses to the last moments of his existence.

It is but rarely that poisonous mushrooms occasion the accidents just described soon after they have been eaten; it is not, most frequently, until after the lapse of five or six, and sometimes twelve, or even twenty, hours that they take place. This seems to dependon the slowness with which these champignons are digested.

The lesions of tissue from them are, numerous and very extensive violet-coloured spots in the skin; great tumefaction of the belly; the conjunctiva of the eyes is injected with blood, and the pupils are contracted. The stomach and bowels are inflamed, and present here and there gangrenous patches: those organs are also strongly contracted, especially the intestines, so that the thickened membranes have entirely obliterated this canal. The lungs are inflamed, and gorged with black blood; the same sort of engorgement exists also in almost all the veins of the abdominal viscera. There are sometimes spots of inflammation, or gangrene, in the membranes of the brain, the pleura, the lungs, diaphragm, mesentery, bladder, uterus, and even in the fetus of a pregnant woman where the mother had been thus poisoned. The blood was very fluid in this woman: it has generally been found coagulated in other patients. Extreme flexibility of the limbs has been sometimes, but not constantly, witnessed.

We shall pass over the author's observations respecting ergot-rye, alcohol, and sulphuric ether, as containing nothing remarkably novel or particularly interesting.

On treating of the effects of sulphureted hydrogen gas on the animal economy, he says that rabbits, ducks, and a young cabiais, died in a few minutes on being confined, with the exception of the head, in bladders filled with this gas. The deleterious effects of it when applied to the surface of the body, appear to be greater in proportion to the smallness of the animal; and a man may remain in a bath impregnated with it for a certain time without inconvenience, provided the gas disengaged is not suffered to enter the lungs by respiration.

The author's observations on the rest of the deleterious gases, and on the effects of the poisons of venomous animals, do not comprise any thing sufficiently novel to require transcription in this analysis of his work.

After having discussed the subjects just mentioned, he treats of poisoning in a general manner, in which he gives only a summary or repetition of what we have already noticed, as far as relates to the signs of poisoning. His disquisition on "the diseases which simulate poisoning," may prove of utility to young students, but we could cite from it nothing very interesting to the generality of our readers. A section follows on the means furnished by chemistry and natural history qualified to render manifest the nature of poisonous substances. This is one of the portions of the work that will not admit of a general abstract.

The author, in the next place, makes some observations on "slow poisoning." The experiments which the author has made on animals for the purpose of elucidating this object, "have proved," he says, "that the accidents produced by very small doses of an energetic poisonous substance, have the closest relation with those which arise from the same poison when administered in a quantity sufficiently great to produce acute poisoning."

The rest of the work, of general interest, relates to "aliments considered in relation to medical police." There is, however, but little that is novel or particularly remarkable adduced on this subject. The author's observations on the adulteration of aliments are either such as we were already made acquainted with, or appertain to local circumstances not sufficiently interesting to English readers to induce us to occupy our Journal with the narration of them. Our object in the review of this work has been to adduce some points which present useful hints to persons already somewhat acquainted with the subjects of it, or which convey some interesting physiological information; not the vain desire of attempting to present in an abstract a satisfactory account of matters that absolutely require minute details in order that they may be applied to their proper purposes. Notwithstanding, therefore, the length of our abstract, we earnestly recommend the book to the attention of students of medical jurisprudence. This, and the work just published by Dr. Gordon Smith, (in which the reader is referred to Orfila's writings for particular information respecting poisons,) will supply them with knowledge that will amply reward the pains taken for its acquisition.

*** *The analysis of the work of Professor* Vacca, *promised in the last Proëmium, is deferred to the next Number, from the want of space for it in the present one.*

